# Cultural probes for environmental education: Designing learning materials to engage children and teenagers with local biodiversity

**DOI:** 10.1371/journal.pone.0262853

**Published:** 2022-02-10

**Authors:** Sónia Matos, Alexandra R. Silva, Duarte Sousa, Ana Picanço, Isabel R. Amorim, Simone Ashby, Rosalina Gabriel, Ana Moura Arroz

**Affiliations:** 1 Interactive Technologies Institute (ITI/LARSyS), Polo Científico e Tecnológico da Madeira, Caminho da Penteada, Funchal, Portugal; 2 School of Design, Edinburgh College of Art, The University of Edinburgh, Edinburgh, United Kingdom; 3 cE3c –Centre for Ecology, Evolution and Environmental Changes / Azorean Biodiversity Group, University of the Azores, Azores, Portugal; 4 Tilburg School of Humanities and Digital Sciences, Language, Communication and Cognition, Tilburg University, Tilburg, The Netherlands; University of Sao Paulo, BRAZIL

## Abstract

Direct contact with nature is paramount in deepening children’s and teenagers’ interest in biodiversity. Learning materials chosen to convey information and engage participants during outings in nature-rich environments are varied and can support rich learning experiences. For this purpose, learning materials can be acquired "off-the-shelf" or developed for site-specific locations or projects. However, there is little guidance on potential techniques for those wishing to generate contextually relevant materials. With the view of responding to this challenge, we propose the cultural probes technique. We demonstrate that the technique, commonly used in qualitative research to generate novel insights in conversation with participants, can instigate innovative and thoughtful approaches to materials designed for children and teenagers to explore nature. We present a toolkit that draws on the literature on cultural probes, inquiry-based learning, and the value of sensory, emotional, and aesthetic experiences in environmental education for structuring interactions with participants. To test our approach, we applied a descriptive research design and mixed-methods approach for collecting questions from youths between the ages of 10 and 18, inspired by a nature walk and a set of exploratory tasks executed through the toolkit. Specifically, we tested our toolkit along a trail in the Nature Park of Terceira, situated in the Azores, a Portuguese volcanic archipelago in the North Atlantic. Here, we present and reflect on the data collected during one visit organized over two days with two groups of participants and one post-trail activity directed at both groups. Results demonstrate that the open-ended and playful nature of cultural probes offers a novel way to engage youths with nature-rich environments through questioning. This contribution further highlights the potential of cultural probes for instigating encounters that tap into the value of sensory, emotional, and aesthetic experience in nature, with positive outcomes for participants.

## Introduction

Environmental Education (EE) strives to create "opportunities to explore nature in the outdoors", to provide "information about conservation and environmental issues", and to convey "knowledge and skills that can be used to defend, protect, conserve, or restore the environment" [1]. To this end, EE also often "encourage(s) different ways of generating meanings of, valuing, conceiving, and contextualizing "nature"" [2]. Paired with EE’s interest in "collective and community learning and action" [3], in this study, we propose the cultural probe (CP) technique as a novel and meaningful approach in EE to engage children and teenagers with local biodiversity.

Drawing on Ardoin et al.’s "exploration of future trends in environmental education research" [3], EE, as a field, includes work from both "natural and social science" and therefore is a "multidisciplinary, interdisciplinary, and transdisciplinary field". Nonetheless, "the two fields that most pre-dominantly engage in EE" are "Education and Communication" [1]. While research produced by EE will often focus on "the integration of EE into formal schooling", the field also contributes knowledge that reflects on "informal settings" [3]. Its contributions also have implications for both the theory and practice of EE. Perhaps, most notably for our project, the field has come to stimulate" (…) researchers to think pedagogically from the student/learner perspective" [3]. As in any field of scientific inquiry, EE "is also continually evolving", whereby "more recent approaches seek to focus on the social dimension of environmental challenges, to more actively address behavior change, and to facilitate rather than lead learning" [1].

Environmental education materials (EEM) can be key in supporting meaningful interaction with nature, both the classroom and in outdoor settings. When developing and designing printed leaflets, books, audiovisual materials, websites, and content for digital devices, it is essential to consider that “materials should acknowledge that feelings, experiences, and attitudes shape environmental perceptions and issues” [4]. For example, EE guidelines, written in support of EEM, highlight the importance of "fairness, accuracy, depth, skills and action building support, instructional soundness, and usability" of materials [4]. Yet, while the literature devises criteria to help those involved in EE select suitable existing EEM [5], there is less support for those wishing to generate context-specific materials.

EE guidance indeed recognizes that materials should “meet the specific needs of the site or community where they will be employed", stating that "content should emerge from and address the needs of a local community" [6]. In fact, “pre-formulated” and “off-the-shelf materials” might not always be efficient and can be replaced by the collaborative creation of tools for education for sustainable development [7]. When working with specific communities, context specificity should reflect the “place” of a community as well as its “identity” or “shared interests” [8].

We developed this study in the Azores, the northernmost of the four Macaronesia archipelagos featuring a Mediterranean hotspot of biodiversity [9], characterized by high levels of endemism [10, 11] and important areas of native vegetation [12], including relict species. Notwithstanding, exotic invasive species (e.g., *Hedychium gardnerianum*, *Pittosporum undulatum*), climate change [13, 14], the building of new infrastructures within protected areas (e.g., roads, geothermal facilities), the degradation and fragmentation of habitats due to human activities such as intensive pastures and urbanization [15], pose significant threats to the conservation of these unique habitats [16].

As a backdrop to the study, we have Field Guide (FG), a project [17] comprising a team of researchers, many of whom are based in the Azores, with expertise in the fields of biology, nature conservation, environmental education, environmental psychology, and human-computer interaction design. The project’s overarching objective is to develop, design, and evaluate mediated modes of learning in natural environments, involving children and teenagers in the Azores. The FG project seeks to provide learning opportunities whilst offering local audiences interactive learning experiences in nature-rich environments through the design of a mobile app created specifically for the project [18]. Rather than opting for “off-the-shelf” content, or generating content a priori for a local audience, we designed this study to better understand if and how biodiversity interests local children and teenagers. In the process, we tested the cultural probe technique, commonly used in qualitative research, and which remains underexplored within the EE literature.

### The cultural probe technique

Design researchers Gaver, Dunne and Pacenti [19] first introduced CP as a qualitative data-gathering technique used to generate inspirational responses in the context of project briefs that aim to design new artifacts. As a research technique, CP "rely on participants’ self-documentation" [20], i.e., information that is, upon consent, later shared with a research team for making sense of the data. For example, in one study [21], researchers used CP to explore "how people live at home" and to ultimately help design researchers "shake the preconceptions" they might hold about the topic. Toward this aim, the same study presented participants with "a disposable camera repackaged with requests for specific pictures" of the home, along with a “friends and family map” to encourage participants to “diagram family relationships”. The study also presented a “Dream Recorder” to facilitate the recording of dreams upon awakening. Data retrieved from CP packages, such as the one described above, often originate subjective interpretation of activities reflecting personal responses arising from participants’ involvement in research.

As detailed above, the CP technique often entails creating packages that present participants with a series of evocative tasks. Packages typically contain maps, postcards, disposable cameras, diaries, and other familiar objects selected for exploring a given context. Thoring et al. [22] present commonly used probe items devised for a CP package, such as a blank notebook for participants to record “observations and ideas”. Maps are also widely used to “structure a given space” where probe activity might occur. The authors also mention “frameworks”, items that help “structure” probe related activities but that are meant for participants to “fill with their ideas or observations” such as “mind maps”, “concept maps”, or “postcards”, to name a few possible examples. Items that capture visual evidence, e.g., through video or photo documentation, are also relevant, along with physical materials that participants can use to develop ideas, depending on the nature of the project.

While clear instructions accompany most probe items, researchers might find it helpful to add “random probes” through which participants can “decide (for) themselves what to document and when”. Akin to quantitative research methods and despite the qualitative nature of the CP technique, the authors also advocate the use of questionnaires and surveys as complements. Finally, “supporting material” in the form of pens and paper is also valuable. At the same time, the inclusion of a “gift” can help make a cultural probe package more compelling and help further encourage participation.

The authors [22] further present probe items according to their function and advise those wishing to use the technique to consider creating or selecting items according to six categories. These include: (1) document observations and activities; (2) envision ideas, wishes, emotions, visions; (3) inspire participation in cultural probe related activity or provide inspiration for the completion of an activity; (4) motivate participation; (5) instruct or explain how probes could or should be used; and (6) items which support a practical function, such as a pen and paper. Whether researchers attribute one or more functions to an item, the authors see the CP technique as an opportunity to stimulate “empathy”, “closeness”, “discussion” and “collaboration” between researchers and participants. CP should also support degrees of “playfulness”, “fun” and “engagement” along with the completion tasks or activities.

In the context of design-led research, Thoring et al. [22] present CP and their potential in: providing "a deeper understanding of a given theme", "making the invisible visible", "inspire design", "foster creativity", and "engender interpretation". Such advantages position CP within a qualitative approach that, at times, can pose challenges as well. As a result, the authors also highlight obstacles encountered in the CP literature. While their list is extensive, here we provide seven challenges that we believe best encapsulate work with CP in the context of EE, namely: "1) finding a balance between information and inspiration, 2) acknowledging uncertainty and ambiguity of probe results, 3) no guarantee for a "hit" with the participants, 4) time for creating a good probe, 5) time for interpreting rich data, 6) participants perceiving probe use as another "job", and (7) “asking in a way that gives you already answers that you know".

### Inquiry-based learning

EEM should not only convey information, but they also “should build lifelong skills that enable learners to address environmental issues” and that emphasize creative thinking skills, such as the formulation of new questions [4]. From this pedagogical perspective, our use of the CP technique in this study is grounded in inquiry-based learning (IBL), since it was our intention to use the CP to elicit responses from participants through questioning.

Informed by a constructivist approach, IBL has mostly impacted the learning sciences with positive implications for environmental education [23, 24]. The ’learning-through-questions’ that forms the core of this approach is particularly valuable. For example, the work of King [25] demonstrates that question-based learning strategies are beneficial in developing critical-thinking and learner autonomy. Concerning learner autonomy, the author notes that “self-generated elaborations have been found to be more conducive to learning then elaborations provided by a teacher, textbook or other external source”. As detailed in the work of Nappi [26], as in the work of King [25], the rationale behind inquiry-based learning, which draws on a Socratic tradition, positions the act of questioning, individually or among peers, as a tool with the potential to prompt a learner to think in new ways and to connect new understandings with existing knowledge. Such a process has implications for developing higher levels of thinking [26] and can yield in greater use of logical and deductive reasoning. Of relevance for the FG project is the argument by Nappi that questioning is essential for our capacity to “make sense of the surrounding world”—a foundational attribute of environmental consciousness [27].

We are accustomed to questioning in science education [28]. However, it is essential to reinforce that ecological literacy also benefits from question-based approaches. For example, the work of Braus and Wood [23] state that "to produce a world of critical and creative thinkers that can help solve environmental problems, we need to encourage students to ask questions and think critically". Similarly, the work of Tolppanen and Aksela [24] highlights the importance of learners’ questions when teaching about climate change. These examples support the idea that environmental education benefits from a learner’s questioning approach. And yet we must distinguish inquiry-based learning from the simple act of questioning, as IBL requires a sustained and reasoned approach to formulating questions [29]. Nonetheless, the IBL literature provides an important pedagogical framework for the design of the toolkit devised for this study.

### The senses, emotion and aesthetic experience in EE

In our use of the CP technique, we also considered the work of Sauvé [30], which identifies 15 currents of intervention in EE. As suggested by the author, these currents are not isolated from one another, and often initiatives will combine the different perspectives outlined in each. What stands out in Sauvé’s review is not only the already established value of cognitive and affective modes of learning, but also the growing importance of sensorial, experiential (e.g., through direct contact with nature), creative (or aesthetic) experiences in EE. Such values reflect the impact of postmodern thought in education and research more broadly, resonating with Lyotard’s view that knowledge is best understood when subjective human experience is equally contemplated [31].

While referencing the work of Jickling [32], Sauvé [30] highlights a perspective of EE that values “sensual experience, sentiments, and emotions, as a way of reconnecting with the rest of the world”. This perspective attunes to more recent work that demonstrates the value of learning in and with the “multisensory richness of landscapes” [27], and of “sensations, perceptions and feelings evoked by aesthetic experiences” in specific biomes [33].

### Research questions

In describing in greater detail the CP technique, IBL and EE’s focus on sensory, emotional, and aesthetic experience, we designed this study with the view of presenting the CP technique to those wishing to generate context-specific materials that engage children and teenagers with biodiversity [7]. In so doing, we demonstrate that CP are not only valid in engaging participants with nature-rich environments through questioning, the technique can also be effective in engaging participants in rich sensory, emotional, and aesthetic ways. Given that when working directly with specific communities (such as island residents) "content should emerge from and address (their) needs" [6], we hypothesize that CP can facilitate the adoption of a participatory approach to the development of topics of interest in conversation with children and teenagers. In addition—and serving as the focus of this study—CP can at the same time provide a meaningful and contextually relevant experience *with* and *in* nature. As a result, we formulated the following research questions:

RQ1: Can the CP technique instigate productive questions regarding the surrounding natural environment on behalf of participants?
RQ2: Can CP provide an outdoor learning experience that taps into the value of sensory, emotional, and aesthetic experiences *in* and *with* nature?
RQ3: Can CP promote participants’ engagement and satisfaction with the activities and with the exploration of nature-rich environments?

## Methodology

### Context

#### Study setting

On the 1st and 7th of December 2019, as part of the activity ‘Rediscovering Nature’, we performed two visits to the trail of Mistérios Negros, located in the Nature Park of Terceira.

**Fig 1:**
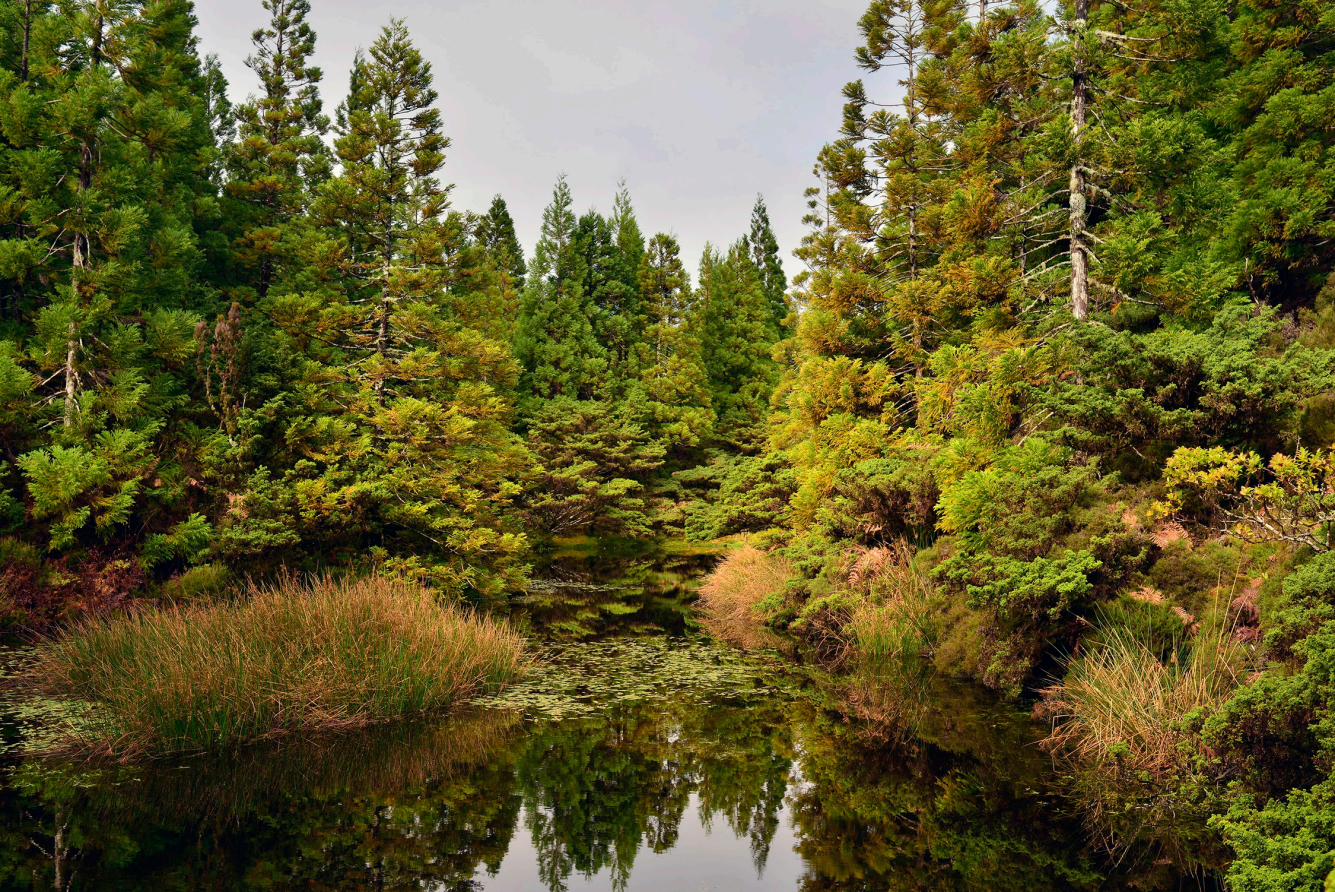
Natural Reserve of Serra de Santa Bárbara and Mistérios Negros—Terceira’s Nature Park.

We chose a trail in a protected area (Natural Reserve of Serra de Santa Bárbara and Mistérios Negros) that would expose participants to native, endemic, and exotic species (Fig 1). Native species arrived in the Azores through long-distance dispersal and therefore exist in other island and continental habitats. In contrast, Azorean endemic species (or subspecies) occur only in the Azores, resulting in either speciation events (neo-endemics) or extinction of the mainland/source populations (paleo-endemics) [10]. The trail also includes exotic species (e.g., *Cryptomeria japonica*), introduced by humans [34]. Some of these species are now classified as invasive (e.g., *Hedychium gardnerianum*) due to their proliferation rate, high abundance cover and negative impacts.

#### Participants

We recruited participants, ages 10 to 18, through the help of two local Scouts groups. Participants and their caretakers accessed information and consent forms via their respective Scouts group. Children and teenagers willingly participated in the study, and we informed them that we might request their participation during a post-trail activity. We also reported to them that they could withdraw their participation at any time. For this study, we obtained ethical approval from the Research Ethics Committee of the University of the Azores.

A total of 36 participants (19 female and 17 male) participated in visits to the trail, coming from both councils of Terceira Island, Angra do Heroísmo (Santa Bárbara) and Praia da Vitória (Lajes). Considering the islands’ small area (400 km2), all residents are relatively close to the Natural Park, less than a half an hour ride, although there is no public transportation for these locations. Of all participants, 23 Scouts were between the ages of 10 and 14, and 13 were between 15 and 18. Most participants (n = 33) joined the reflective post-trail activity performed at the university a month and a half later.

Regarding participants’ exposure to local biodiversity, we note that teachers working with the Azorean primary and middle school curricula can adapt content and materials to the region’s socioeconomic and environmental context [35]. This flexibility offers teachers space to include subjects concerning local Azorean biodiversity, the region’s protected areas and its context-specific environmental issues. However, because the curricula are flexible, it is difficult to know with certainty and depth which Azorean living organisms, protected areas, and environmental issues students explore in class. However, following our communication with the Directorate of the Natural Park of Terceira, only 10.4% of the islands’ students visited the park during the period previous to the COVID-19 pandemic, 2018–2019 [36].

#### Data collection methods

In this study, we first developed a CP toolkit with the Azorean natural forest ecosystems in mind. We then implemented the toolkit during a trail walk with local children and teenagers. We collected data based on questions formulated by the participants and their final impressions from the trail walk. Later, we conducted a post-trail activity in a classroom setting, where we asked participants about their willingness to return to the trail and why.

For its construction, we adopted the six categories commonly used applying the CP technique, as documented by Thoring et al. [22]: (1) "document”, (2) "envision", (3) "inspire", (4) "motivate", (5) "instruct", and (6) "support". We describe the toolkit, in greater detail in the following sub-section. Approximately two months after the trail walk, we invited participants to a follow-up activity at the University of the Azores. By adopting a descriptive research design, and mixed-methods approach that made use of surveys and observations for collecting both quantitative and qualitative data, we were able to assess the CP’s:

1. Efficacy in promoting productive questioning for the future development of EEM (RQ1).
2. Effectiveness in promoting sensory, emotional, and aesthetic experiences in nature (RQ2).
3. Potential to positively engage youth with nature-rich environments (RQ3).

As detailed in Table 1, we assessed productive questioning by looking at the ratio between the questions motivated by a need to obtain an answer (and therefore obtain or confirm knowledge), and questions formulated to satisfy a request (i.e., in response to the toolkit’s suggestion that participants pose a question following each activity). While the first group corresponds to productive questions, the second group corresponds to strategic questions. Here, it is important to note that we do not treat the questions individually as such although they are essential to the FG project in signaling themes we may wish to act upon in the subsequent development of the project.

**Table 1:** Analytical model of the CP technique’s efficacy.

Constructs	Dimension	Indicators
Productive questioning	Type of questions	Type of approach to the task of questioning
Experiences in nature	Sensory, emotional, and aesthetic experiences	Dominant theme of the final responses
Engagement with nature-rich environments	Satisfaction	Degree of the evaluative judgments of the final responses
		Willingness to return to the trail
	Motivation	Semantic field of the motives of satisfaction of the final responses
		Focus of the motives to return to the trail

Secondly, we assessed the efficacy of the technique in promoting sensory, emotional, and aesthetic experiences in nature-rich environments by looking at the frequency with which participants, in the final free, open-ended activity, focused their responses on qualities experienced during the trail walk (e.g., concerning a specific location, a moment, a feeling, etc.).

Finally, we assessed participants’ engagement with a nature-rich environment by analyzing: the observations registered by adult volunteers during the trail walk; the participants’ evaluative judgments expressed in the final free, open-ended activity (to assess their degree of motivation and satisfaction with the trail walk); and participants’ willingness and motivation to return to the trail.

#### The field guide cultural probe toolkit

**Fig 2:**
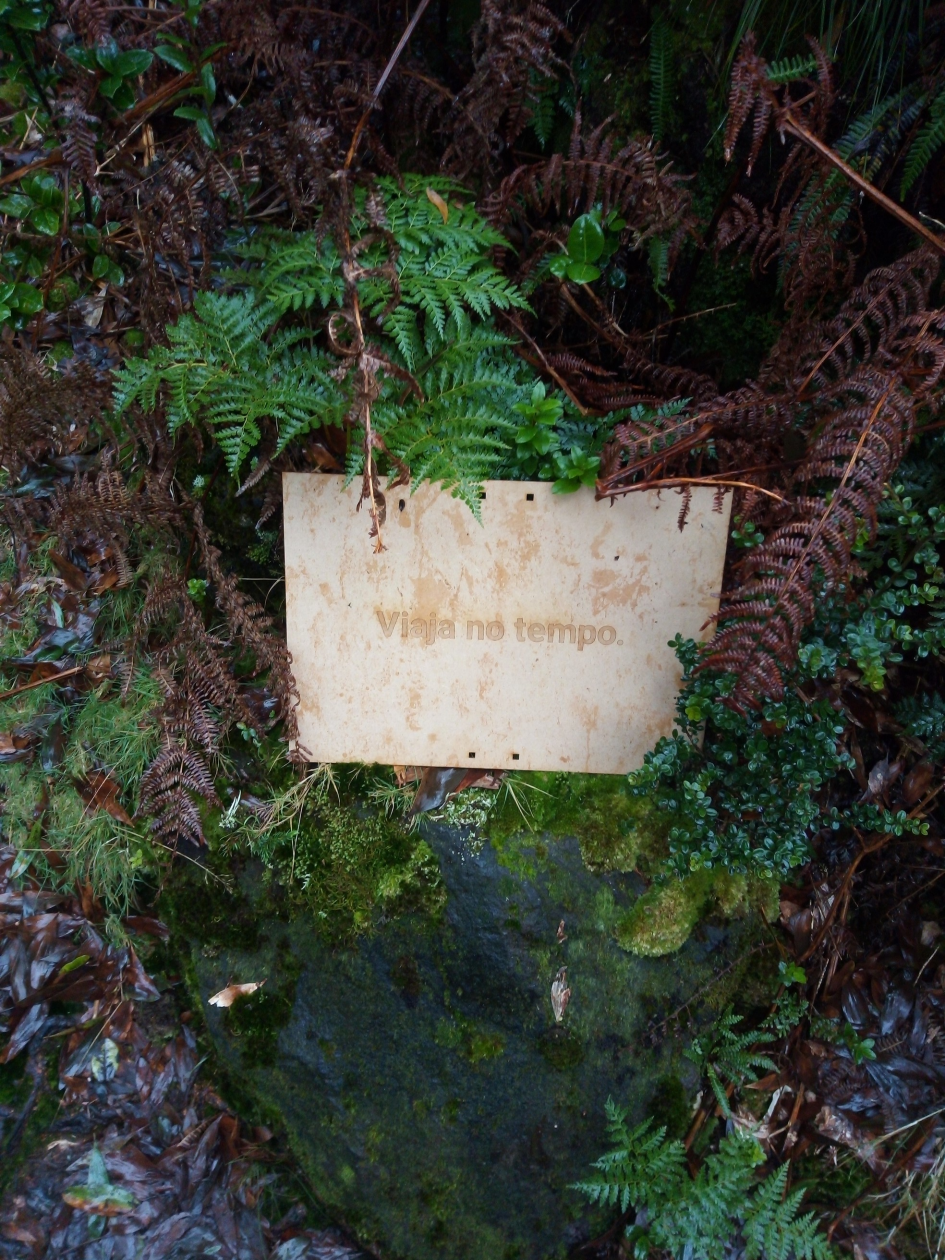
Station sign displaying the original Portuguese title ‘*Viaja no Tempo’* (‘Travel in Time’).

**Fig 3:**
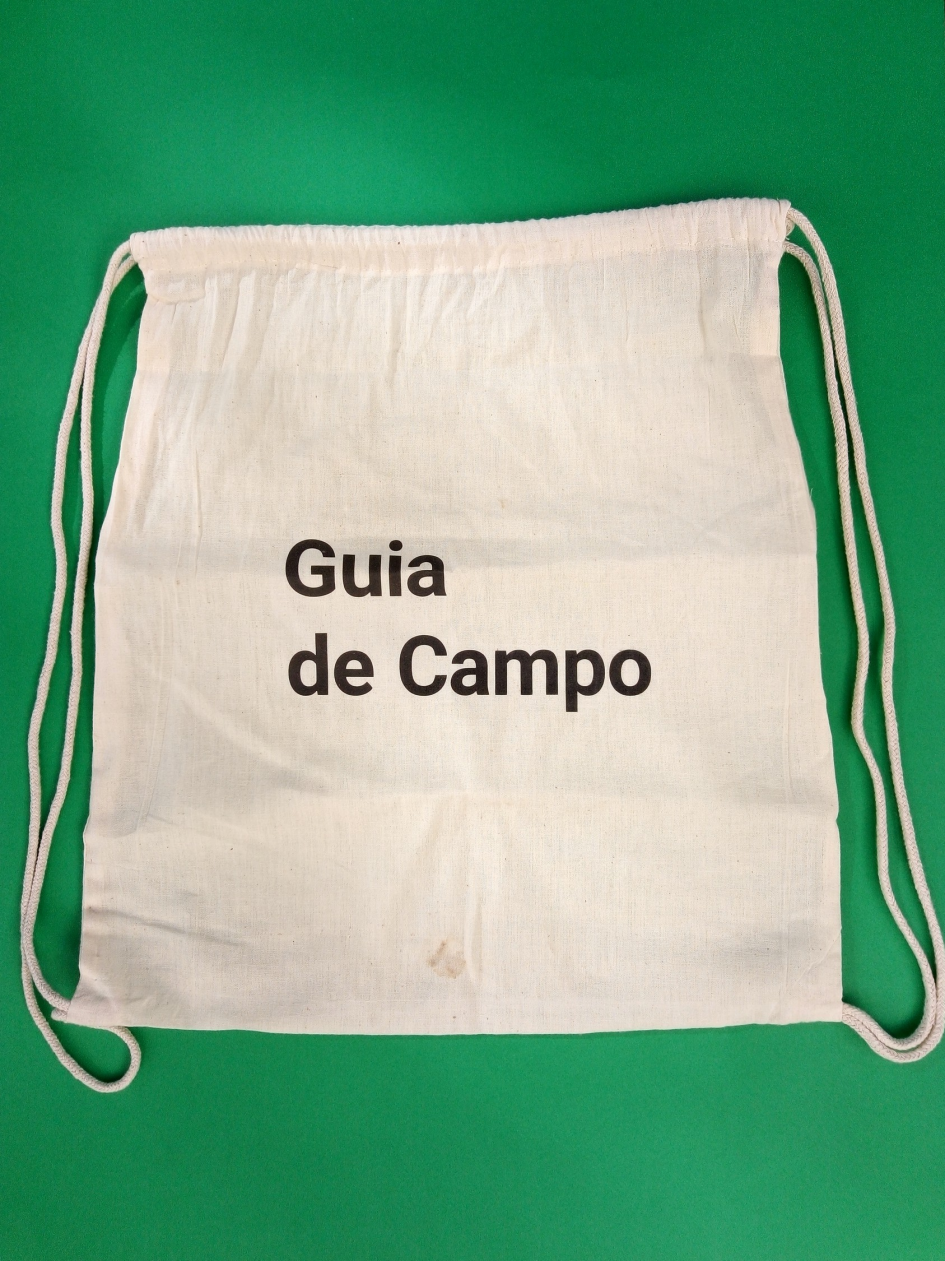
The Field Guide cultural probe toolkit canvas bag (with original project title in Portuguese).

**Fig 4:**
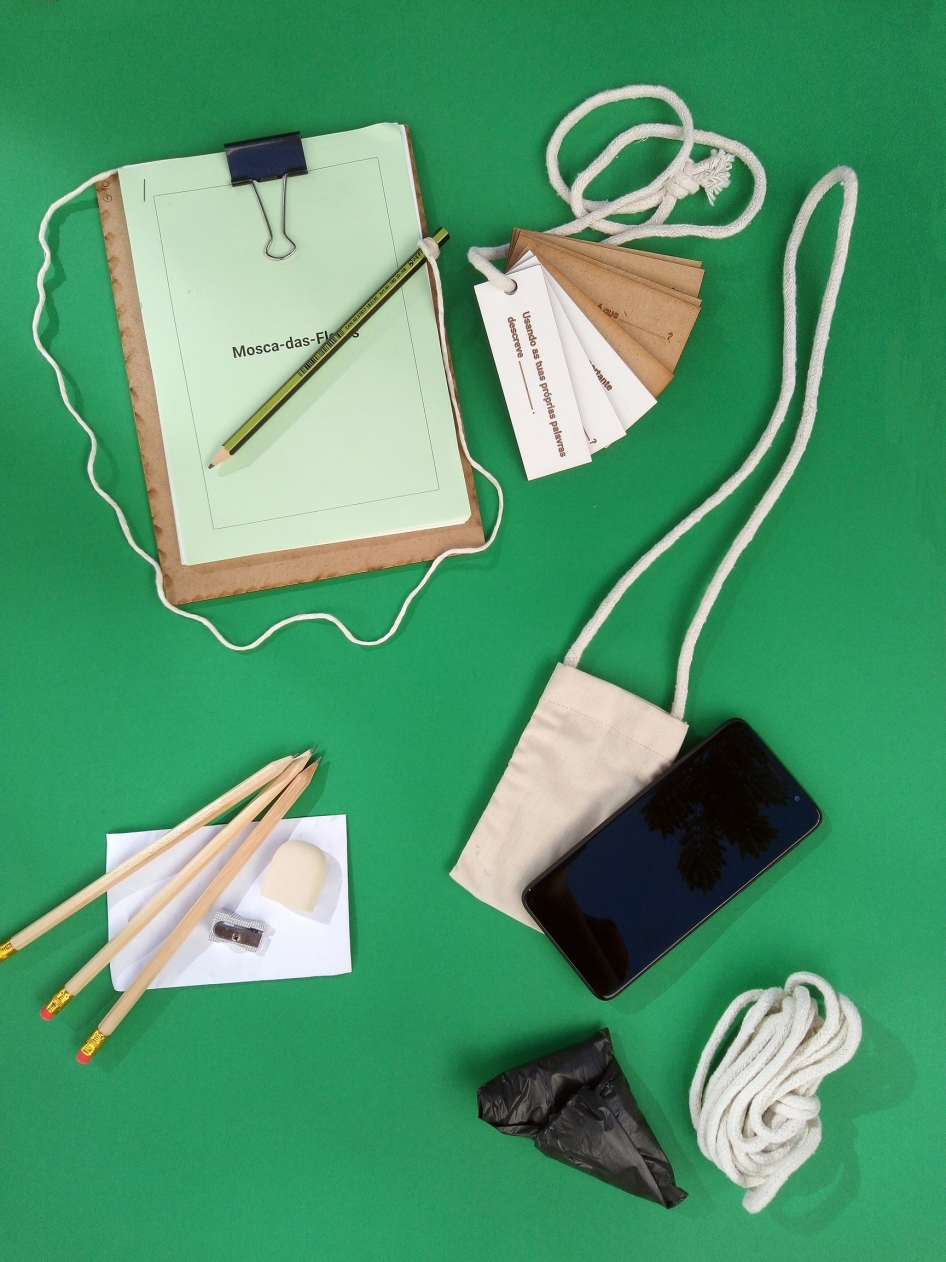
Toolkit bag contents.

**Fig 5:**
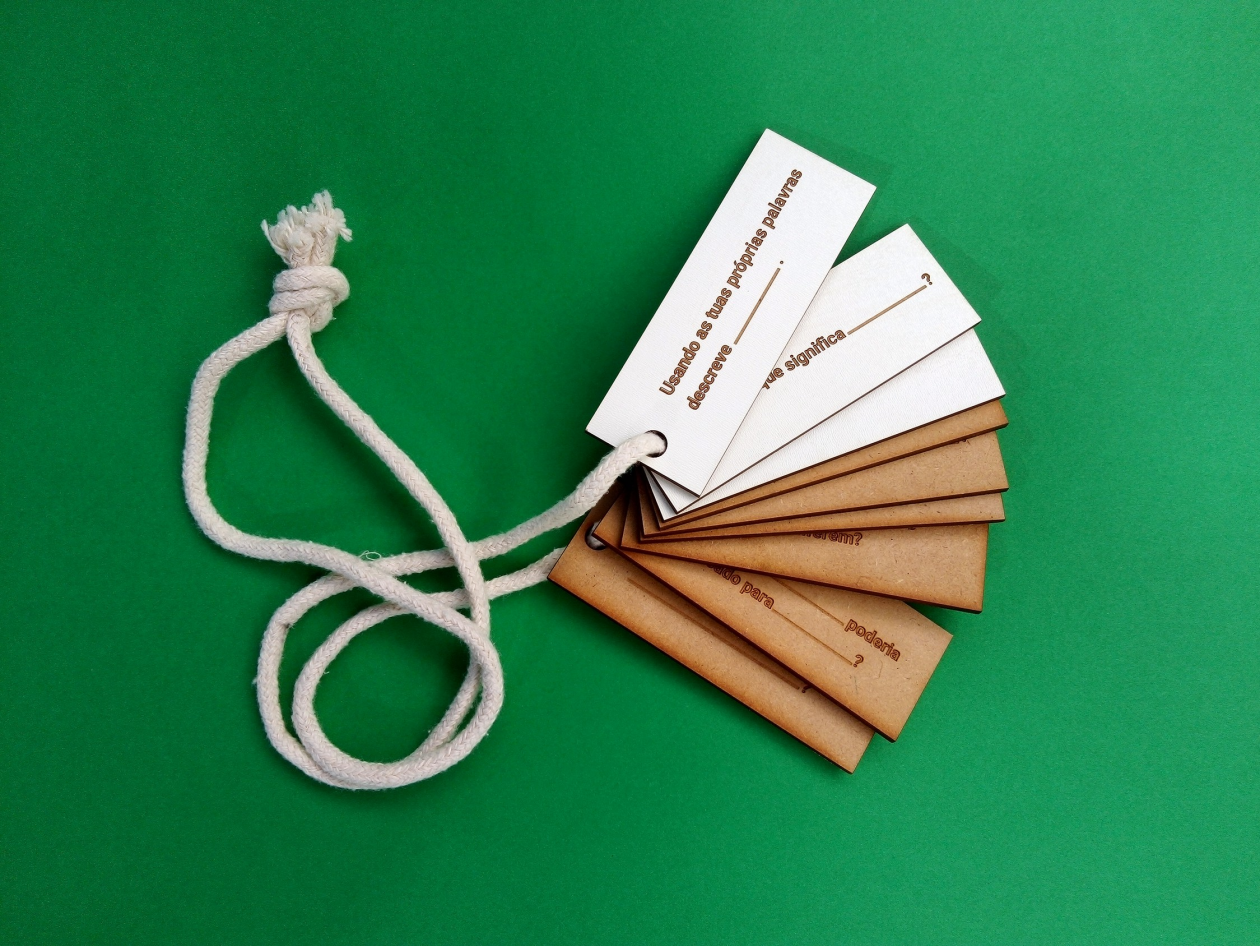
Question tokens (with questions in Portuguese).

Figs 2, 3, 4 and 5 present the FG cultural probe toolkit with signs demarcating station points along the nature trail. Figs 3 and 4 show a cotton canvas bag that was specially designed for the project to contain and transport probe items.

We divided the first two kilometers of the trail into 12 demarcated stations and added beginning (Station 1) and endpoints (Station 14) to provide an opportunity to understand participants’ impressions of the surrounding environment. At the beginning of the trail, participants were invited to perform a free-word association exercise in response to a given sentence; at the end, participants were asked to briefly close their eyes for a moment and then write down any words that came to mind (Table 2). In line with Thoring et al. [22], we designed both beginning and end stations for documentation, inspiration and motivation. Stations 2 to 13 bore a title expressed with a single word or short phrase (Table 2, in bold). As in Gaver et al. [19, 21], we intentionally thought of creating signage as a means of defining specific locations on the trail and eliciting curiosity, therefore aligning with Thoring et al. [22] with respect to the of capacity CP to inspire participants. In each station participants were asked to engage with a specific activity designed to both inspire interaction with nature and instruct on the activity itself.

**Table 2:** Station titles and their corresponding activities.

Station 1. **Take a break.** Before we start our walk, close your eyes for a moment and then write the words that come to mind.
Station 2. **The mystery of water.** Squeeze these two plants (moss and Japanese cedar) and measure the resulting volume of water. Observe the differences.
Station 3. **The smells of the forest.** Identify as many smells as you can. Indicate the ones you dislike as well as the ones you like the most.
Station 4. **Help the forest.** This plant is one of the invasive species that takes up most space from the original plants of the Azores. You can help control this problem by pulling out a plant or, by removing all the leaves.
Station 5. **Create your park.** Use the cord provided in your pack to delimit an area (‘reserve’) that contains what you think is most important to keep. Photograph your ‘reserve’, give it a name, and record the time when the corresponding photo was taken.
Station 6. **Who lives here?** Select the three species that you consider to be most characteristic of this site and photograph them. Discuss the reasons why you chose these species and what you know, or would like to know, about each one.
Station 7. **Where am I?** Discuss and record your answer.
Station 8. **Touch and feel**. Describe everything you feel as you touch mosses, ferns, and tree trunks.
Station 9. **Manager for 10 min.** Identify 4 elements that exist in this lagoon. Of those that do not exist, choose the ones you would like to see here while referring to what would change with their introduction to the lagoon.
Station 10. **Where are the animals?** Look for animals on various substrates: air, water, soil, bark, etc. List them and quantify them.
Station 11. **Hear the Forest.** Be silent until you hear the bell and make a list of the sounds you identified. List: (1) what you heard; (2) what you heard and were not expecting to hear; (3) what you were waiting to hear and did not hear. Now read the poem below by Alberto Caeiro (here translated by the leading author from the original version in Portuguese): That lady has a piano Which is nice but it’s not the running of rivers Nor the murmur the trees make… What do you need a piano for? It’s best to have ears And love nature.
Station 12. **Lime-Green-Bud-Green.** Take photographs of the different shades of green you see here. Now imagine what this landscape would look like without some of these shades. What if you could only see one shade of green here? Discuss these questions.
Station 13. **Travel in time.** Imagine going back 250 years in time. You are in the year 1769, eight years after the eruption of Mistérios Negros in 1761. Describe what was here at the time. Now travel 250 years into the future. You are in the year 2269. Describe what you see.
Station 14. **Close your eyes.** We have reached the end of our walk. Once again, close your eyes for a moment and then write the words that come to mind…

As shown in Fig 4, the toolkit bag contained practical items, such as an A5 clipboard, three pencils, an envelope with a pencil sharpener and an eraser, a cord for completing the ’Create your park’ activity, and a trash bag for the ’Help the forest’ activity. We provided activity sheets for each station to document participants’ responses to activities when relevant, and to inspire interaction with the surrounding environment. We also included one mobile phone per group with a protective pouch to document the ’Create your park’, ’Who lives here?’ and ’Lime-Green-Bud-Green’ activities. A set of wooden tokens (Fig 5) featuring the content-free questions served the purpose of motivating participants to ask questions and instructing them on possible types of questions.

In line with the inquiry-based educational literature that emphasizes the positive benefits of devising support strategies for question-based learning [37], we designed nine medium-density tokens, laser-printed on fiberboard (Fig 5) and etched with a content-free question (Table 3). Our intention was to facilitate the formulation of participant questions through content free questions. Nine of the 10 content-free questions draw on King [38], as translated to Portuguese. However, we excluded one of King’s questions—"How does … tie in with …. that we learned before", given the lack of a formal learning activity prior to the visiting the trail. Participants also had access to an additional question sheet for each station, except for the start and endpoints. With this sheet, we hoped to document the questions formulated by participating groups with the help of the tokens.

**Table 3:** Content-free questions.

1. Describe … in your own words.
2. What does … mean?
3. Why is … important?
4. Explain why …
5. Explain how …
6. How are …. and …. similar?
7. ’’How do… and… differ?’’.
8. How could … be used to …?
9. What would happen if …?

While we employed an IBL strategy in the design of question tokens, the toolkit was designed to explicitly evoke sensory experiences by inviting participants to attune their senses to nature through sensation such as touch, feel, squeeze, smell, and hear, as indicated in Activities 2, 3, 8, and 11 (Table 2). With respect to emotion, station titles, such as ’Help the forest’, were intended to elicit in participants a sense of care for nature. Instructions asking participants to pause, be silent, hear, smell, touch, visually admire different natural elements were designed to encourage an aesthetic experience. We also invited participants to engage introspectively with nature while reading a poem by Alberto Caeiro (one of Fernando Pessoa’s heteronyms) [39] in Activity 11 (Table 2). Individual stations additionally provided more standard information regarding local nature conservation. See, for example, Activity 4, ’Help the forest’ (Table 2), where we present the kahili ginger (*Hedychium gardnerianum*), an invasive species that poses a significant threat to biodiversity in the Azores [40].

To summarize, in Table 4, we present the FG CP toolkit according to the categories and functions described by Thoring et al. [22].

**Table 4:** Field Guide cultural probe toolkit, according to the categories and functions described by Thoring et al. [22].

Item	Category	Function
Station signs	Maps	Inspire, Instruct
Cotton canvas bag	Wrapping	Practical
Activity sheets	Frameworks	Inspire, Instruct, Motivate
Question sheets	Frameworks	Document
Content-free question tokens	Frameworks	Instruct, Visionary
Mobile phone	Photo/ Video Documentation	Document
Drawing board, three pencils, pencil sharpener, and eraser	Supporting Material	Document
Cord, trash bag	Supporting Material	Practical, Motivational

#### Procedure

We performed two visits on the 1st and 7th of December 2019. In the first visit, we welcomed 15 participants (eight between the ages of 10–14 and seven between 15–18). In the second visit, we welcomed 21 participants (15 between the ages of 10–14 and six between 15–18). Due to the length of the trail, the level of difficulty, and the time needed to cover the terrain, younger participants (ages 10–14) visited Stations 2 to 7, the older participants (ages 15–18) visited Stations 8 to 13, this in addition to Station 1 and 14 which both groups visited.

Before initiating the trail activity, we asked participants to perform a free-word association exercise in response to the sentence (here translated from Portuguese): "Write freely the words that come to mind when you think of ’natural forests of the Azores‴. With this exercise, we wanted to gauge participants’ impressions of the local natural environment we were about to visit. At the end of the trail, we asked participants to close their eyes for a moment and then write down any words that came to mind.

With the support of 10 adult volunteers during both visits, we welcomed participants to the starting point where they established themselves into groups of two or three members. We followed with an explanation of the activity. Group members were encouraged to adopt different roles, such as note-taking, transportation of the canvas bag, transport, and mobile phone use, while it was explained that the posing of question prompts was a group task. At this point, we also presented the canvas bag and its contents.

It is important to note that adult volunteers contributed alongside the research team in the form of structured notetaking, involving: (1) sensory impressions, (2) observations regarding interaction and communication, and (3) any additional notes. For the sensory impressions category, we looked for data relating to participants’ mood in each station (excitement, boredom, etc.). For the interaction and communication category, our focus was on the interaction between participants in each group. For example, how did participants delegate tasks and share resources? Was there a good synergy between group members? Was anyone left out? For the additional notes category, we asked volunteers and participating team members to record extracts of sentences or conversations uttered between participants.

#### Data analysis

We transcribed data collected during the trail walk and the post-trail activity using Microsoft Excel software to synthesize and categorize data. A thematic analysis of the data was necessary to define the categories of the indicators (type of approach to the task of questioning, focus of the motives to return to the trail, dominant theme of the final responses; and degree of the evaluative judgements of the final responses). Themes were developed a posteriori, without a pre-existing categorization system. This inductive process included all responses collected over the two visits. We expand on the operationalizations of the different categories in the Findings section.

To ensure the interpretation and validity of the results, we used several quality processes, namely:

1. Triangulation: two researchers with different academic backgrounds (one with an MS in Biology and one with a PhD in Psychology) performed the analysis.
2. Intercoder reliability test: a Cohen’s kappa coefficient was calculated in a random sample of 35 of the 237 units of meaning, categorized independently by four judges, and re-calculated after redefining categories to solve disagreements and until we reached a near-perfect agreement result (0.98).
3. Iterative data processing: performed to include all relevant information from participants as well as accommodate new refinements of the classification system for the only category that resulted in an initial poor agreement kappa coefficient (0.20; "type of approach in the task of questioning" category).

To analyze participants’ degree of satisfaction with the trail walk, we also submitted their responses to the final free, open-ended activity to a similarity analysis of the semantic field. After translating the responses from Portuguese to English, we used the freeware program IRAMUTEQ [41] that produces a maximum tree of words by grouping the words and expressions into communities and interconnecting the communities to each other according to the frequency of evocations and the number of co-occurrences [42]. To explore differences between age groups, we performed Pearson’s Chi-square tests (χ2), with a 95% confidence level (p ≤ .05), using the IBM SPSS Statistics for Windows software Version 27 [43]. When verified that more than 20% of expected frequencies were below five; we used the likelihood ratio value (G2) to replace Pearson’s Chi-square value (χ2).

## Findings

### RQ1

- Can the CP technique instigate productive questions regarding the surrounding natural environment on behalf of participants?

By the end of the trail walk, we retrieved 168 questions. Four were incomplete, making it impossible to categorize them. From the remaining 164 valid questions, 137 were found to be sufficiently distinctive, indicating a high level of diversity of questions posed. Following an inductive process, we analyzed questions with respect to their related activity and the supervisor’s comments concerning participants performance in each activity. As a result, we were able to rank valid questions as either ’productive’ or ’strategic’.

The ’strategic’ category corresponds to questions triggered by the invitation to complete the study’s tasks or by design of the activity itself. In these cases, participants overlooked the opportunity to obtain new information through questioning by deriving questions based on those presented on the tokens. For example, we received the question "Describe the sounds of nature", which is similar to the question posed to participants during the activity at Station 11: "(…) make a list of the sounds you identified". Note that our use of the term ’strategic’ differs from King’s [25], who in one study used the word to define questions "used by students to guide their planning and monitoring activity during problem solving".

In the ’productive’ category, we included internally oriented questions triggered by the desire to question and obtain knowledge or confirm previous knowledge related to the external context. This category comprises externally oriented questions triggered by participants’ exploration of the external context, such as the natural context, or the previously performed activities. In either context, ’productive’ questions are those that refer to a genuine interest in knowing more.

**Fig 6:**
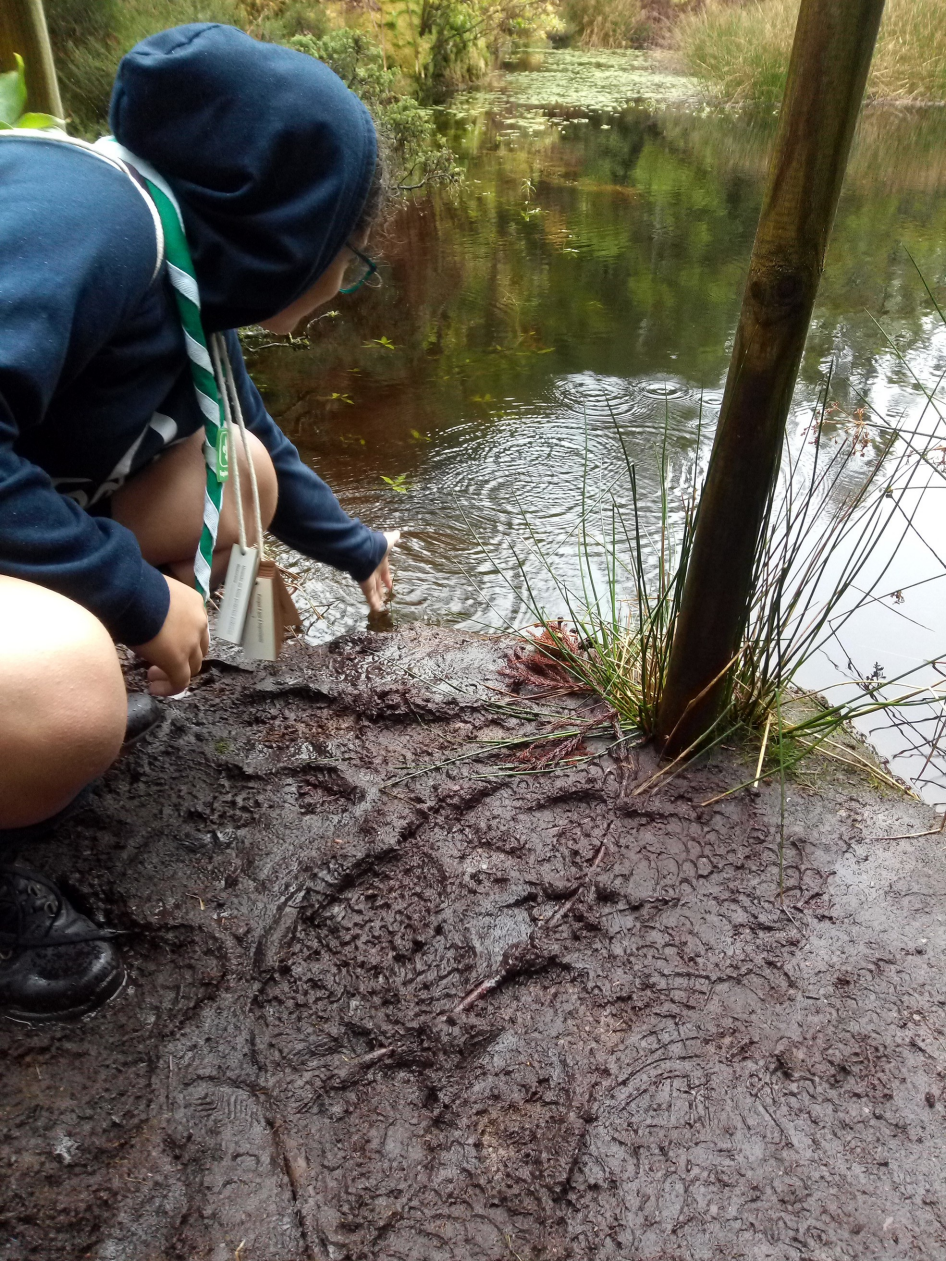
One participant touches water of the lagoon that can be found on the trail.

An example of an internally oriented question is "Why is biodiversity important?" which does not directly accrue from the activity performed in Station 3 ("The smells of the forest"). Also, a comment from the station supervisor suggests that the group who produced this question was suitably motivated ("They are doing the work with certain dedication."). An example of an externally oriented question is ‘‘How could the water from the lagoon be used?”, which, although it does not directly relate to the corresponding activity, relates to the natural context of Station 10 (‘‘Where are the animals?”), where it was produced. Note that this station was also near the lagoon that can be found in Mistérios Negros (Fig 6).

**Fig 7:**
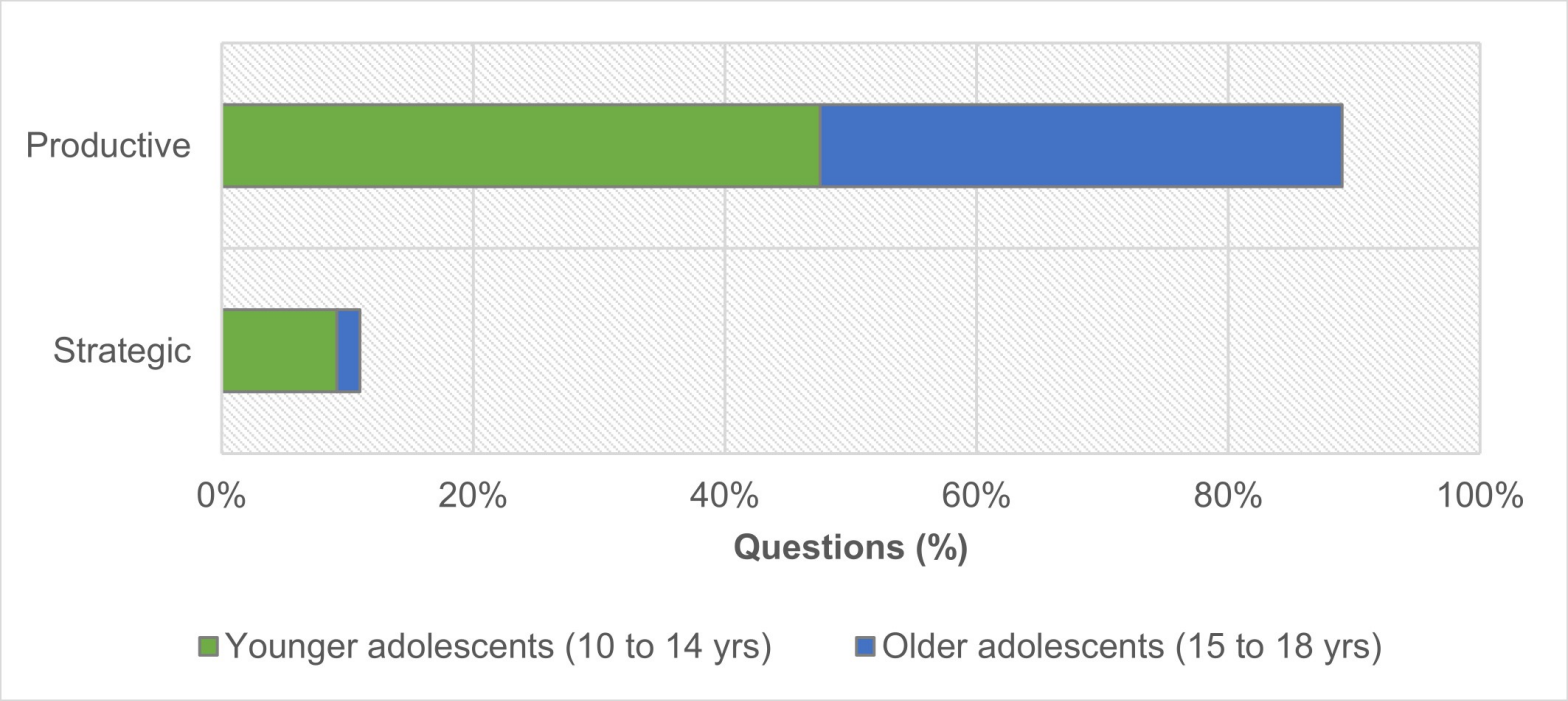
Distribution of valid participant questions (n = 164) according to the approach adopted in questioning by age group. In total, younger participants generated 93 questions, and older participants generated 71 questions.

From the 164 valid questions, nine-tenths (89%; n = 146) were found to be ‘productive’, while nearly all the questions were externally oriented (94%; n = 137), focusing on the immediate natural context or performed activities. Only 11% (n = 18) were categorized as ‘strategic’ questions (Fig 7). The collected data suggest that the CP technique promoted productive questioning concerning the surrounding nature-rich environment.

**Fig 8:**
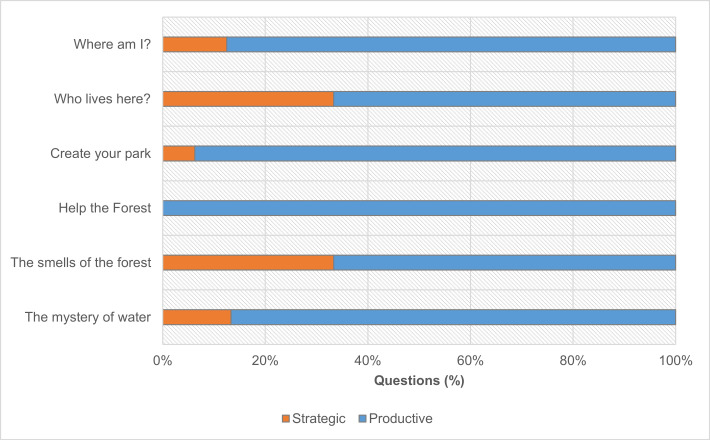
Distribution of ‘productive’ and ‘strategic’ questions (n = 93) of younger participants (ages 10–14) according to the stations where they were formulated (the total number of questions produced in each station varies between 15 and 16).

Overall, differences between age groups are significant (χ2(1) = 5.839; p = .016), and the number of ’strategic’ questions is much smaller in the older group. Nonetheless, both groups generated a higher number of ’productive’ questions; 84% (78 out of 93) for the younger participants, 96% (68 out of 71) for the older participants. Additionally, we crossed the questions posed with their corresponding activities to determine whether some activities prompted more ’strategic’ questions than others. For the younger participants (Fig 8), the distribution of ’productive’ and ’strategic’ questions by activity is not homogenous (χ2(5) = 12.666; p = .027).

**Fig 9:**
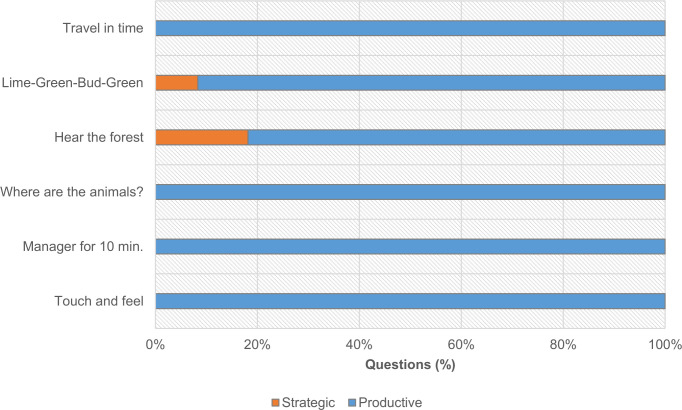
Distribution of ‘productive’ and ‘strategic’ questions (n = 71) of older participants (between the ages of 15–18) according to the stations where they were formulated (the total number of questions produced in each station varies between 11 and 12).

Activities 3 ("The smells of the forest") and 6 ("Who lives here?") raised a more significant number of ’strategic’ questions when compared to other activities. For the older group (Fig 9), ‘strategic’ questions represent only three out of 71, corresponding to just two activities. Thus, the distribution of ‘productive’ and ‘strategic’ questions was not significantly different between activities (χ2(5) = 7.541; p = .183).

**Fig 10:**
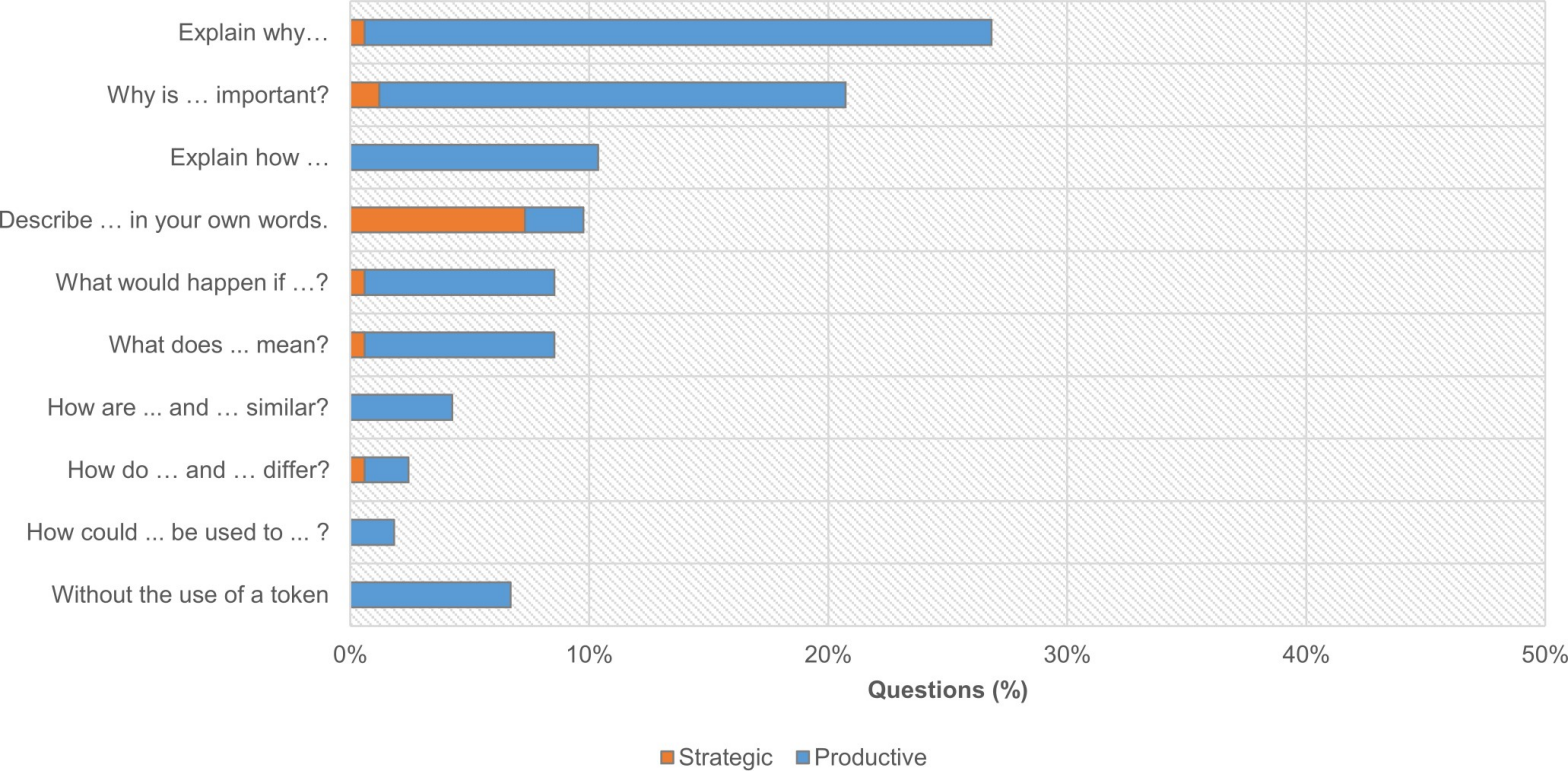
Distribution of ‘productive’ and ‘strategic’ questions (n = 164) according to the question tokens used in their production.

When distributing questions by question tokens used in their production (Fig 10), we observed that participants used all of the tokens provided. However, their selection is not homogenous; it is evident that the tokens ‘‘Explain why…?” (27%; n = 44) and ‘‘Why is… important?” (21%; n = 34) were preferred. In the first token, the focus is on possible causes–e.g., ‘‘Explain why invasive plants exist?”–and the purpose of something–e.g., ‘‘Explain why nature needs moss, fern, and trees?”. In the second token, the focus is on the relevance of the purpose–e.g., ‘‘Why is preserving nature important?”.

Tokens related to comparison, e.g., "How are… and… similar" and "How do… and… differ", as well as those associated with the utility of something, e.g., "How could… be used to…?" were used less often. We further observed that only 7% of the questions (n = 11) were constructed without the use of tokens–e.g., "Do plants choose their natural environment?". Thus, content-free question tokens appear to benefit participants’ questioning. Additionally, the distribution of ’productive’ and ’strategic’ questions is significantly different between question tokens (χ2(9) = 51,828; p < .001). The token "Describe… in your own words" promoted more ’strategic’ questions than the other tokens. Three-quarters (12 in 16) of the questions produced with this token were found to be ’strategic’, possibly owing to the descriptive nature of the token that encourages the production of questions by adding just one noun.

### RQ2

- Can CP provide an outdoor learning experience that taps into the value of sensory, emotional, and aesthetic experiences *in* and *with* nature?

From the last free, open-ended activity, we retrieved 34 responses (two participants did not respond), which were then translated from Portuguese to English. From the analysis, we observed that 22 participants responded with free-word associations (e.g., ‘‘silence, singing, birds”) and 12 with phrases (e.g., ‘‘This journey was magnificent with beautiful plants and landscapes”).

We categorized all 34 responses according to their "dominant theme", or the main idea expressed when attributing meaning to the experience. Responses highlight participants’ perspectives that arise from features of the activity, with our interpretation of the "dominant theme" closely following Litfin’s [44] definition of a "frame" of an object or phenomenon. Following an inductive process, and after analyzing all possible answers, we identified three main thematic categories. We infer that participants focused their responses on one of the following:

1. The “experience of the natural context”.
2. The “assessment of the activities”.
3. The “description of the immediate context”.

Responses categorized under (1) ‘‘experience of the natural context” are those in which the answer, or the dominant tone, of the answer emphasizes qualities of the lived experience in the natural context. Even when it includes descriptive elements, the description is usually associated with the perception of the natural environment through the senses or feelings. For example, ‘‘nature, joy, happiness, beauty” (Table 5) emphasizes sentiments aroused through the experience of the nature-rich context.

**Table 5:** Illustrative responses from the final free, open-ended activity according to three types of ‘‘dominant themes”, inspired by Litfin [1995].

Dominant theme	Examples of responses
Experience of the natural context	‘‘beauty, delight, rest, smell”
	‘‘nature, joy, happiness, beauty”
	‘‘Nature is the best that we can have because with her we can feel her sounds and fresh air, that is the contrary of the city.”
Assessment of the activities	‘‘This activity was interesting because I was able to pay attention to details and ask questions that I had never thought of before.”
	‘‘With this activity we were able to learn a little about some concepts of nature and about existent and non-existent animals in the lagoon or on the island.“
	‘‘I really liked the project. I think it is good that we can help in this kind of creative activity.”
Description of the immediate context	‘‘nature, birds, river, pond, scouting”
	‘‘rain, wind, mud, grass, islands, handkerchief”
	‘‘trees, plants, wood, squirrels, scoutmaster, moss”

Responses categorized under (2) ‘‘assessment of the activities” correspond to answers whereby the answer itself, or the dominant tone, focuses on assessing the trail walk and/or the activities that it comprises. E.g., the participant response ‘‘This activity was interesting because I was able to pay attention to details and ask questions that I had never thought of before”.

**Fig 11:**
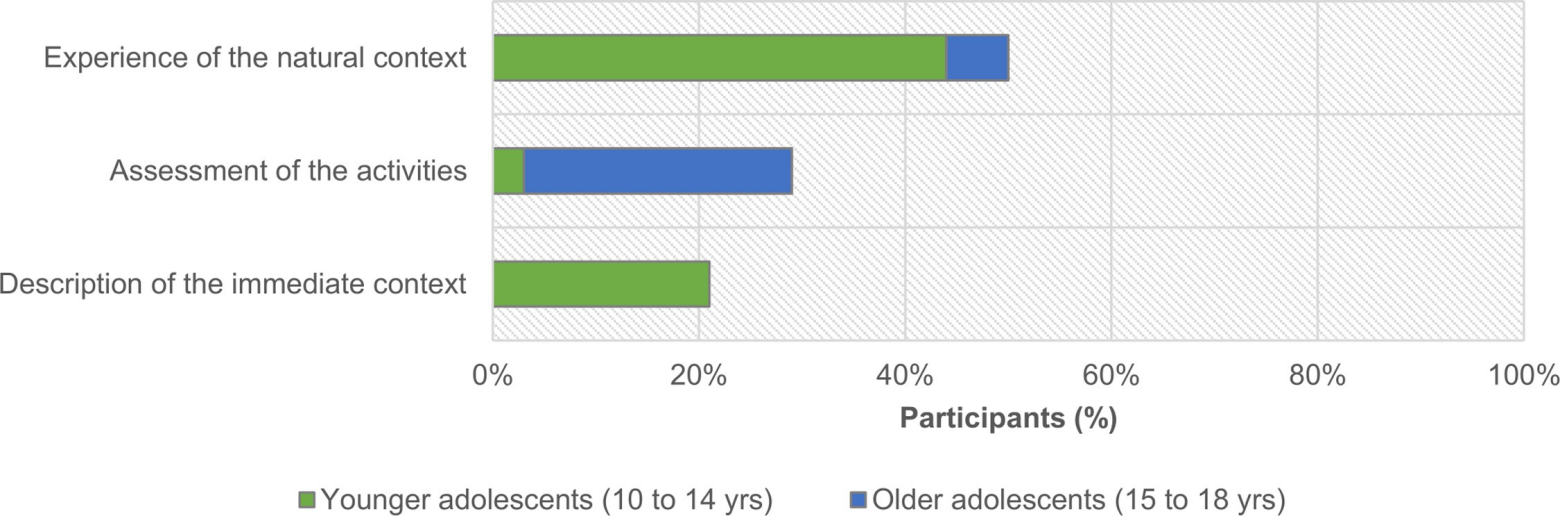
Distribution of participants (n = 34), according to age group, by the dominant theme of their responses to the final free, open-ended activity.

Those categorized under (3) ‘‘description of the immediate context” correspond to responses which enumerate, in an emotionally neutral way, elements of the participant’s direct experience. For example, ‘‘nature, birds, river, pond, scouting” describes only the features present in the external context (natural or not). Results also show that almost half of the participants (47%; n = 17) emphasized experiences *in* and *with* nature, as recollected during the open-ended activity. Less than one-third (28%; n = 10) focused their responses on the assessment of the activity itself, while one-fifth (21%; n = 7) described the immediate context (Fig 11).

Differences between age groups show that the assessment of the activity is more frequent in the older group, while younger participants represent the totality of descriptions of the immediate context, and the generality of the experiences relating to the natural context (Fig 11; χ2(2) = 23,989; p < .001).

The general prevalence of aesthetic, sensory, and emotional experiences in the final free, open-ended activity (signaled through words such as "beauty, delight, rest, smell") suggests that the CP offered an outdoor learning opportunity that builds on participants’ experience of nature. Moreover, we believe that experiences lived during the study might have been recognized by more participants if we had asked them to do so directly. Nonetheless, the indirect way in which responses were elicited gave us greater access to the relevance and authenticity of their experience. The open-ended nature of the questioning further allowed for assessing participants’ direct awareness of their experience.

### RQ3

- Were the participants engaged with the CP? How did the CP promote their motivation and satisfaction in natural-rich environments?

All participants completed the proposed activity tasks, suggesting that they engaged with the activities, despite a certain awkwardness displayed by a few participants towards some of the more hands-on activities (as communicated in the notes from adult volunteers), e.g., when asked to touch wet vegetation.

Furthermore, comments collected by volunteers in each station indicate that participants showed "focus and commitment", a positive attitude ("good synergy between them") and were responsive to all tasks proposed at each station. The volunteers also observed that participant groups engaged in conversations focusing on performed activities or the natural context. These seem conducive to questioning, as demonstrated in the extracts of dialogues between participants collected by volunteers: "What is peat?", "It’s an animal?", "Who lives here?", "Why do invasive species harm the forest?", "What can we use moss for?". Notes were also indicative of curiosity among some groups regarding natural phenomena ("Why do invasive species harm the forest?"). Additionally, the notes taken by adult volunteers suggest that younger participants showed "satisfaction and a positive attitude", while indicating that those participants were also more "enthusiastic" than the older ones.

**Fig 12:**
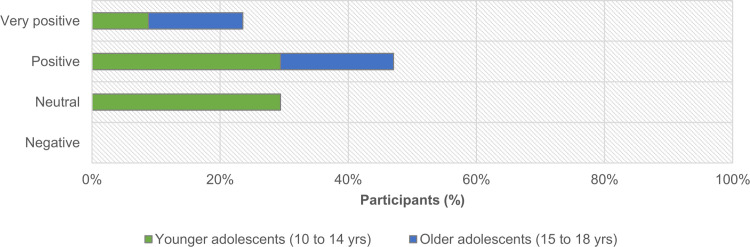
Distribution of participants (n = 34), according to age groups, by the degree of the evaluative judgements expressed in their responses to the final free, open-ended activity.

At the end of the trail, in the activity proposed in Station 14, participants’ comments and evaluative judgments regarding their experience on the trail reveal a high degree of satisfaction with the activities and their exploration of the natural context. After an inductive process, we categorized the degree of satisfaction as neutral, positive, or very positive (Fig 12). ‘Neutral’ comments were those in which participants described only the immediate context without any evaluative judgment (e.g., “nature, hiking, forests”). ‘Very positive’ comments differed from ‘positive’ comments in the use of superlative adjectives (e.g., ‘‘most beautiful”, referencing landscapes, weather, etc.’), and adverbs that emphasize the verb (e.g., ‘‘I *really* liked the project and the landscapes were very beautiful.”). ‘Positive’ comments involved favorable explicit evaluations (e.g., ‘‘This activity allowed me to get to know nature more closely and to know how to better appreciate my surroundings”), or favorable implicit evaluations (e.g., ‘‘birds, life, love, peace”, and ‘‘This activity made me look at nature differently and that we must preserve our surroundings”).

Results show that responses did not reflect negative evaluative judgments. More than two-thirds of participants (71%; n = 24) expressed ‘positive’ or ‘very positive’ evaluative judgments, with the older ones always positively appreciating the trail walk (Fig 12; χ2(2) = 11,051; p = .004). In the positive or very positive comments, we did not find significant differences within dominant themes–the activities and the experience of the natural context (χ2(1) = 0,341; p = .559).

**Fig 13:**
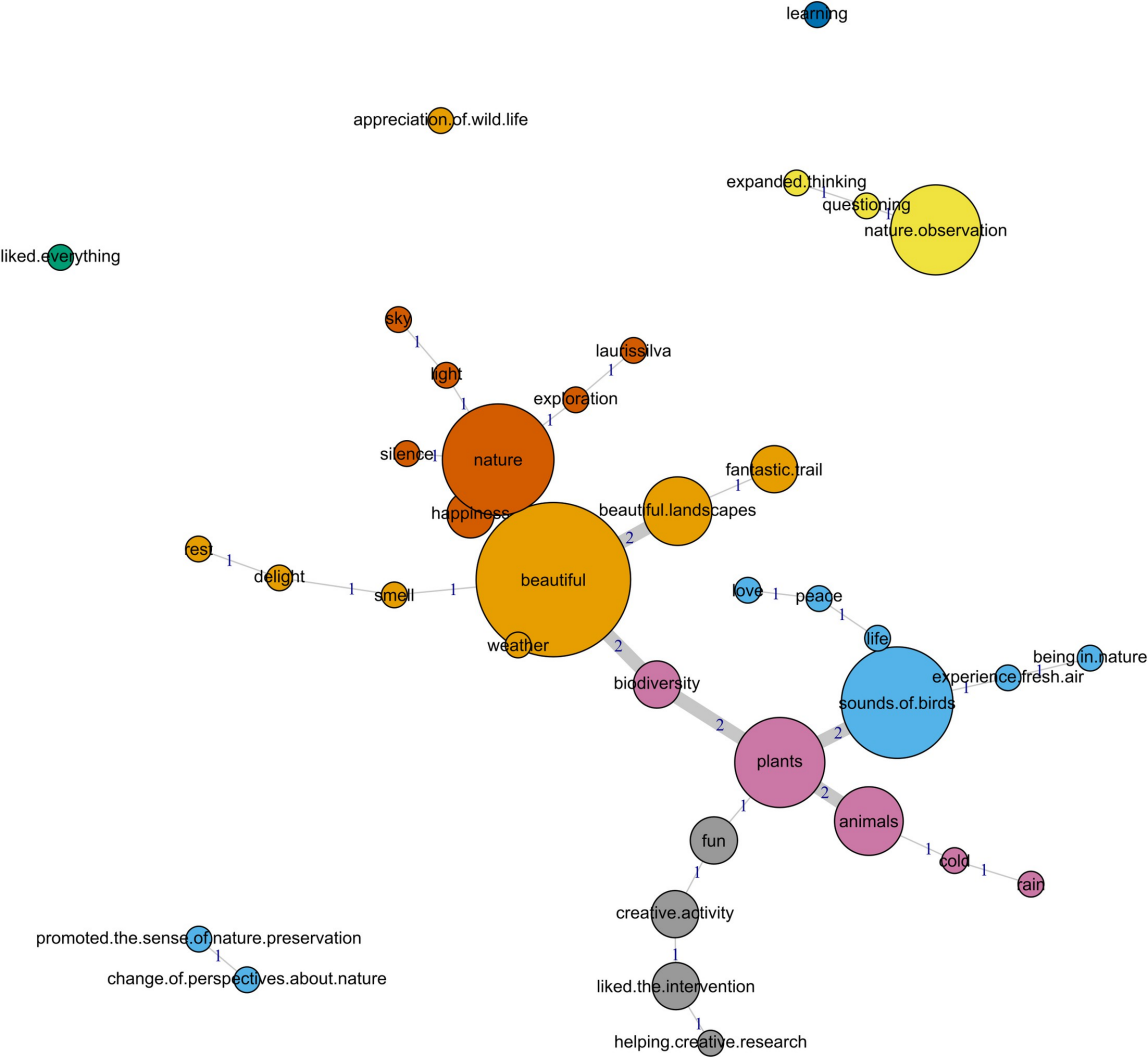
Results of the lexical similarity analysis representing the relationships between the 34 responses to the final free, open-ended activity. Line thickness and numbers correspond to the frequency of co-occurrence, circle sizes to evocation frequency, and each color aggregates clusters of meaning.

To understand participants’ satisfaction levels, we analyzed the similarity of the semantic field for both ’positive’ and ’very positive comments. The resulting maximum tree (Fig 13) shows a very expressive cluster (orange group) where the pleasantness of nature’s aesthetics predominates ("beautiful"; "beautiful landscapes"; "delight"; etc.). Blue and dark orange clusters, on the other hand, reveal a respective focus on the experience of nature ("sounds of birds"; "experience fresh air"; "peace"; etc.) and the natural context itself ("nature"; "laurissilva"; "silence"; etc.), involve multiple senses and sensations. The pink cluster also reveals a focus on the natural context, but with more attention to the specific elements perceived. In the remaining three clusters, participant satisfaction was focused on the trail walk itself, its qualities ("fun"; "creative activity"; etc.) and outputs ("nature observation"; "expanded thinking"; change of perspectives about nature"; "promoted the sense of nature preservation"; etc.).

The willingness to return to the trail also constitutes an indirect indicator of participant satisfaction when exploring the nature-rich environment. Apart from three of the 36 participants who were absent from the post-trial activity, all remaining participants expressed a willingness to return to the trail.

**Fig 14:**
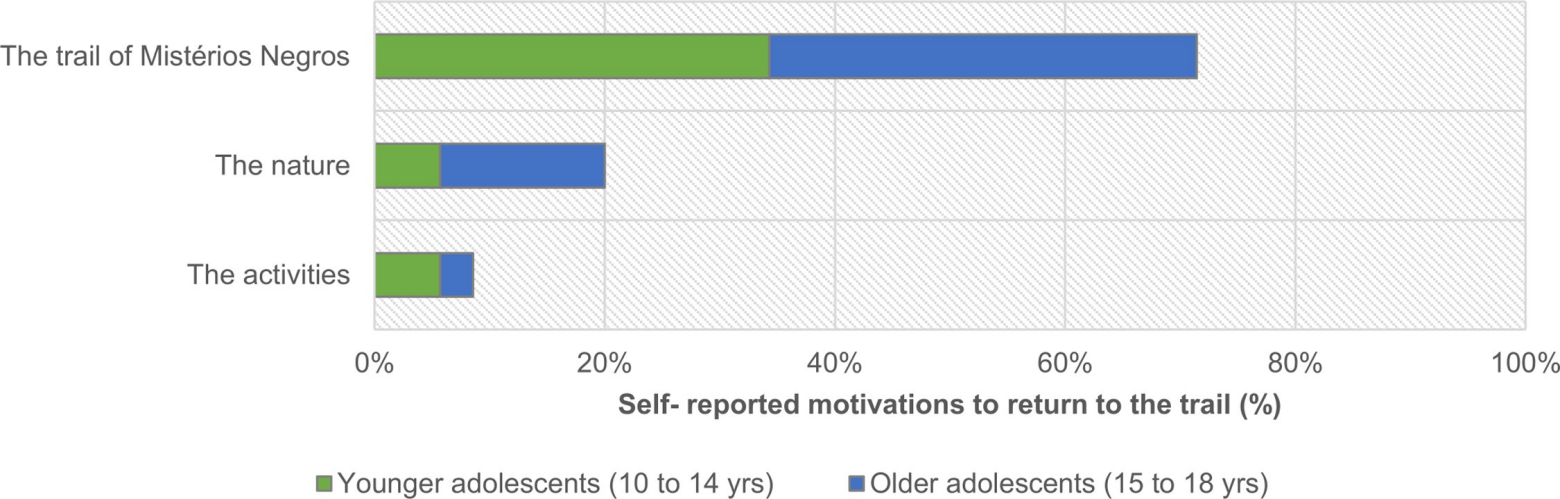
Distribution of self-reported motivations (n = 35) to return to the trail, collected in the post-trail activity from the 33 participants, according to focus and age group.

From an inductive analysis of the 35 valid self-reported motivations to return to the trail (Fig 14), we identified three primary motivations: the activities, the nature, and the trail of Mistérios Negros itself. Some participants expressed arguments that focus on their enjoyment of the trail walk and its activities (e.g., ‘‘Because I liked the experience.”; ‘‘It was fun”), others focus on the experience of being in the outdoors (e.g., ‘‘Because I love hiking, exploring and nature”), while still others focus on certain characteristics of the trail itself (e.g., ‘‘Because it is an interesting trail”).

From the data analysis, we can observe that 71% (n = 25) of motivations focus on the trail itself, 20% (n = 7) on the natural context, and 9% (n = 3) on the activities performed during the trail walk, with no significant differences found between age groups (Fig 14; χ2(3) = 4,867; p = .118).

## Discussion

Our results suggest that the CP effectively promoted participants’ engagement with a nature-rich environment during visits to the trail of Mistérios Negros. Nonetheless, we conclude that the way a CP toolkit introduces questioning in proposed activities can influence participant responses. During analysis, the need to avoid a saturation of question forming within each activity became evident as an essential consideration in the design of IBL-inspired CP. For example, the task associated with Activity 6 already presupposed questions. When asked to write two more questions, the fatigue may have resulted in participants adopting a strategic approach with the view of quickly concluding the activity. Question prompts should then be subtle and well-integrated when questioning is part of an activity. In addition, the ages of participants appear to positively influence the generation of productive questions, although for the current study, we are unable to draw a more robust conclusion regarding age, considering that the activities were different for each group.

Nonetheless, it was clear that overall, some tokens produced more strategic questions. Also, tokens were not equally significant in generating questions concerning nature. Tokens prompting comparisons ("How are… and… similar?", and "How do… and… differ?") and those relating to the utility of something ("How could… be used to…?") were less used. It is possible that such tokens limit the expressive power of participant-generated questions, compared to the other tokens. For example, the question "How do trees and mosses differ?" was created only by adding two nouns to the question. The token that asks participants to identify a concrete purpose or utility for something resulted in questions such as "How could the water from the lagoon be used?". In this instance, participants did not use the complete token, possibly considering that it was not easy to find a concrete purpose when contemplating the utility of the water in the lagoon. Although we have no evidence to suggest how our participants might favor ’doing’ versus ’knowing’ (procedural knowledge/experiential learning), we speculate that a question token that requires focus on the utility of something can pose challenges.

Results gathered in response to RQ1 shed light on the application of IBL in the design of learning materials for EE. Drawing on the broader literature, as in the work of Lazonder and Harmsen [37], questioning is effective when supported. However, as detailed above, there are various ways to support inquiry. The study we describe here relied on question tokens. Given the authors’ categorization of support as "unguided, minimally guided, (or) guided", and the data obtained in this study, question tokens only "minimally guided" participants in posing questions. As a result, we believe that more consistent guidance would be a necessary precursor for achieving better results in the quality and quantity of productive questions generated. This also begs a new research question: what level of guidance in questioning activities can CP provide in the context of place-based learning in EE? Productive questioning benefits from using question tokens as proposed in our study. Still, we need more data that would allow us to compare different ways of integrating question guidance within a CP toolkit. For example, although tokens facilitated the production of questions and did not significantly constrain participants, future studies should include testing that assesses the output of questions with and without tokens, along with a participant debrief aimed at exploring participants’ perceptions on the utility of such tokens.

Considering "the impact of culture on inquiry" [29], designing a CP toolkit within the context of EE would benefit from a more comprehensive integration of IBL within participants’ broader learning experiences, both within and outside the school environment. Lack of studies on the use of IBL in the Portuguese curriculum [45] might not necessarily entail a lack of questioning and inquiry-related experiences on behalf of children and teenagers enrolled in the Portuguese school system. Nonetheless, using a CP toolkit for EE that is grounded in IBL could provide an opportunity through which questioning, and ultimately inquiry, are introduced to children and teenagers.

In response to RQ2, we can infer that the CP toolkit effectively promoted experiences grounded in sensory, emotional, and aesthetic qualities in a nature-rich environment. Nonetheless, and considering the data collected in response to RQ1, we noticed some discrepancies. Activity 3, for example, raised more strategic questions than productive ones. We believe that this could express the general prevalence given to sight or sound in Western culture. Furthermore, and aligning with the broader literature, e.g., Stevenson et al. [46], we speculate that as children and teenagers do not often explore smell, this activity raised more strategic than productive questions. Rather than avoiding working with multiple senses in designing a CP toolkit for EE, we believe the contrary; this information highlights the opportunity to use EE to expose children and teenagers to various sensory experiences in nature-rich environments. A CP toolkit could be an ideal potential technique for achieving this.

We further noticed that the experience of the trail, as guided by the CP toolkit designed for this study, was very present in the participants’ awareness of the trail walk. However, there are age differences, with older participants tending to make more assessments, while younger people focused more on experiencing and describing the external context. We believe this reflects differences in child and teen learning, which are affected by changes in behavior, cognition, and the brain [47], as well as the effects of different types of schooling, which impacts students in different ways [48]. This information feeds back on the type of activities and their suitability for the different age groups. It also begs further research for uncovering how to best use CP in the context of EE, and when designing for children versus teenagers.

Finally, and in response to RQ3, the CP toolkit designed for this study promoted participants’ positive experiences of a nature-rich environment, such as the trail of Mistérios Negros. We believe that the design of the various station activities influenced this outcome. They provided information regarding different elements, such as the native, endemic, and exotic species that characterize the trail, along with geographical information, such as the lagoon. Station activities also engaged participants through their senses, providing a springboard for imaginative exploration, as observed in response to the activities of Stations 11 and 13.

We also understand that working with a protected area with pristine nature was paramount to participants’ experience of the CP toolkit. Exposure to nature positively impacts our mood and sense of general well-being [49]. While the FG project focuses on the appreciation of Azorean biodiversity, we believe that data obtained in response to RQ3 begs further testing of the CP technique in what Saari and Mullen [50] have termed ’dark places’. Such places require a shift from our view of nature "from pristine, ’natural’ places in place-based pedagogy to including their crossings and layerings with urban, industrial and polluted spaces"—Saari and Mullen referring to Garrard [51]. Such places are as important in the promotion of biodiversity conservation as pristine ones. They might generate a host of different thoughts and experiences on behalf of children and teenagers that are nonetheless relevant. Of course, this does not exclude the value that nature-rich environments provide to humans and how their impact on physical and psychological well-being is vital to their conservation [49].

The CP literature, concepts drawn from IBL and the value of sensory, emotional, and aesthetic experiences in EE were paramount in the design of an appropriate CP for FG and the present study. Nonetheless, we derive certain key practical implications that support the creation and application of CP in the context of EE, as follows:

1. Create probes that balance information regarding nature-rich environments with opportunities to experience nature through imagination and the senses directly.
2. Ground the design of CP in relevant pedagogical framework, such as inquiry-based learning.
3. If using IBL, consider participants’ experience with questioning and inquiry, and devise probes that support prior experience or lack thereof.
4. Provide genuine opportunities for feedback without saturating participants with requests for information through questioning. In this instance, subtle integration is key.

### Limitations and future work

Our study was limited in time and participant numbers. We recruited participants through two local Scout groups, thereby assembling a convenience sample even though participant demographics align with the project’s target audience: local school-aged children and teenagers (ages 10 to 18). In addition, when conducting the study, we did not have prior information regarding participants’ knowledge of the local environment. Despite this, as we walked the trail, notes from the research team suggest that even though exposure to the outdoors is crucial to Scouts’ activities, participants appeared to know little about local and native species and had little experience on nature trails. For most, this was their first visit to the trail.

Furthermore, we were aware that word association exercises could pose challenges. The qualitative nature of the activity demands a capacity on the part of researchers to interpret participants’ subjective responses. However, we were also aware that such exercise could elicit more deep-seated and personal interpretations of the activity which are valuable for future design [52]. We were confident that the trade-off would be worthwhile. As well, we did not want to risk breaking the flow of the activity with a post-activity questionnaire or interview. Thus, the creation of stations with accompanying exercises designed to encourage free-word association was deemed optimal. Nonetheless, we recognize that supplementary reflection on the activity could further substantiate some of our claims.

Continuous exposure to the CP technique in the context of EE is essential to draw more robust conclusions regarding all three research questions. Despite this, we can say in hindsight that a question-based strategy provided research participants with a rich experience during data-gathering. We retrieved a high number of questions (168 in total), suggesting that questioning could be a rich topic for the discussion of CP within the context of EE.

## Conclusion

The purpose of this paper was to present a research method to EE; in doing so, we experimented with the CP technique, a qualitative research strategy that holds potential for involving a local community of participants. Our goal was to understand what interests’ children and teenagers as they interact with nature-rich environments. Akin to the early “community content-based instruction” model [6], we intend to use the results to aid in the subsequent design of materials for the FG project. Additionally, and in line with the CP literature [19, 21], this study was an opportunity to build a rapport with research participants who also represent a local community of children and teenagers. We achieved this by designing a CP toolkit that affords an informal learning experience in a nature-rich environment.

We were keen to use the toolkit to invite participants to share their curiosity in interacting with the surrounding natural world with our research team. Preliminary results suggest that a CP toolkit containing question-based activities can afford positive meaningful experiences *in* and *with* nature.

By situating this study’s findings within EE research, we hope that the unique characteristics of CP can be understood and utilized by the EE community. Although these strategies are born out of design-related inquiry and qualitative research more broadly, our analysis shows that we can apply CP to create EEM. However, further research is needed to support our argument and better implement the CP technique within the range of contexts that characterize contemporary EE.

## Supporting information

S1 Dataset (XLSX)

S1 Fig (TIF)
