# Supplementary figures and images for: Cultural probes for environmental education: Designing learning materials to engage children and teenagers with local biodiversity
Source: PLoS One. 2022 Feb 10;17(2):e0262853. doi: 10.1371/journal.pone.0262853 (PMC8830674; doi:10.1371/journal.pone.0262853)

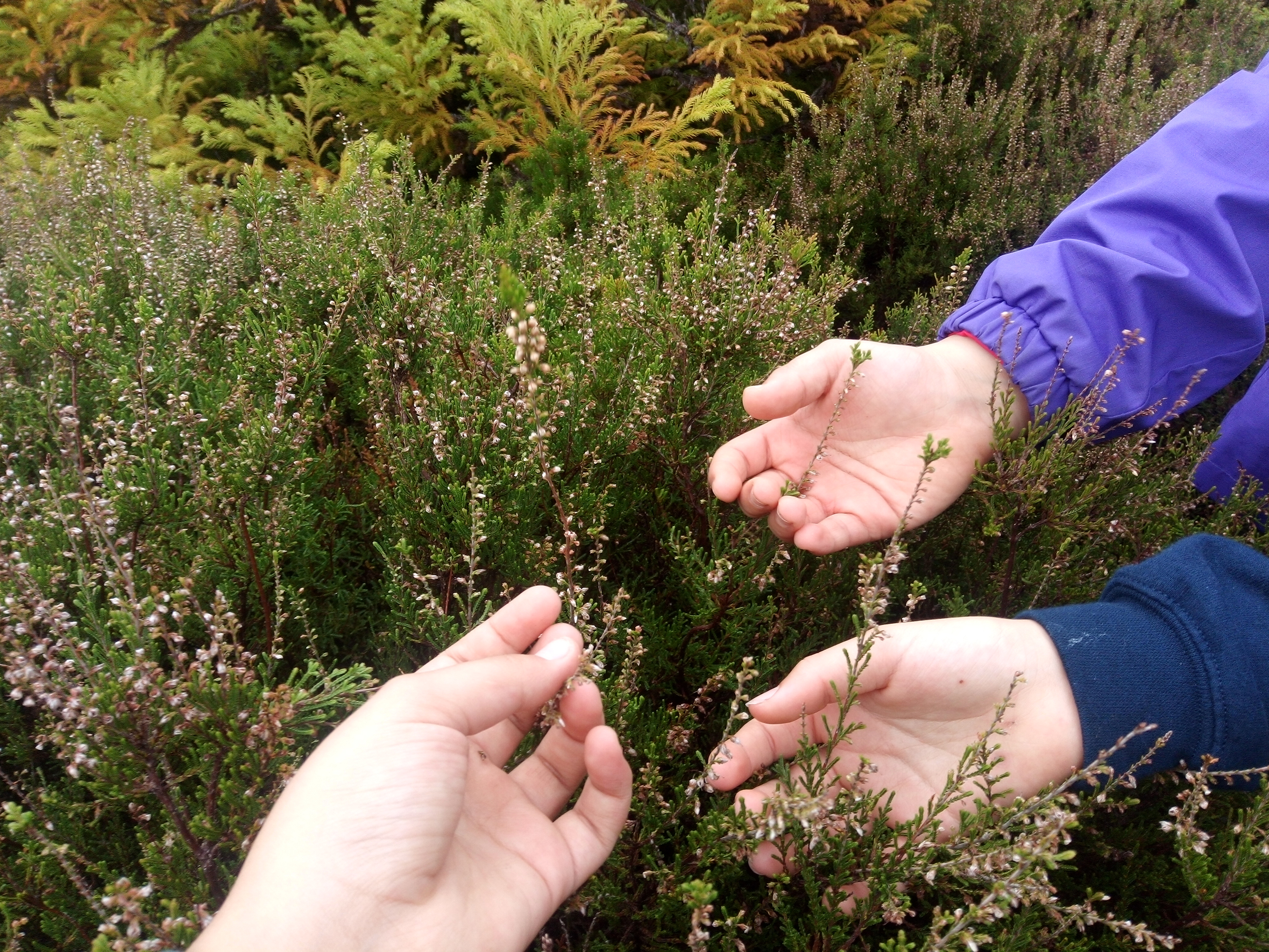

Supplement: S1 Fig — (TIF) [file pone.0262853.s002.tif]
